# Israeli spotted fever rickettsia (Rickettsia conorii complex) associated with human disease in Portugal.

**DOI:** 10.3201/eid0506.990620

**Published:** 1999

**Authors:** F Bacellar, L Beati, A França, J Poças, R Regnery, A Filipe


other is a small clinical and epidemiologic study of hospitalized children <2 years of age in Santiago de Compostela (8). Data from both studies are consistent with our results. Rotavirus gastroenteritis is a common cause of hospitalization and produces a heavy load on the health-care system in our region. After years of research, vaccines that effectively prevent rotavirus infections in humans have been developed (9,10). These data should be considered in evaluating the potential benefits of introducing rotavirus vaccine in our region and monitoring its effectiveness.

### Acknowledgments

We thank Maribel Mendiburu and Antxon Nuñez for their valuable assistance.

This study was supported in part by a grant from the "Fondo de Investigaciones Sanitarias de la Seguridad Social" (Spanish Ministry of Health and Consumption), FIS 92/0612.

**G. Cilla G, E. Pérez-Trallero, L.D. Piñeiro, A. Iturzaeta, and D. Vicente**

Servicio de Microbiología, Complejo Hospitalario Donostia, San Sebastián, Spain

### References

1. Glass RI, Bresee JS, Parashar UD, Holman RC, Gentsch JR. First rotavirus vaccine licensed: Is there really a need? Acta Paediatr 1999;88 Suppl 426:S2-8.
2. Johansen K, Bennet R, Bondesson K, Eriksson M, Hedlund K-O, De Verdier Klingenberg, et al. Incidence and estimates of the disease burden of rotavirus in Sweden. Acta Paediatr 1999;88 Suppl 426:S20-3.
3. Ryan MJ, Ramsay M, Brown D, Gay NJ, Farrington CP, Wall PG. Hospital admissions attributable to rotavirus infection in England and Wales. J Infect Dis 1996;174 Suppl 1:S12-8.
4. Ministerio de Sanidad y Consumo. Clasificación internacional de enfermedades. Novena revisión. Modificación clínica, 1994.
5. Hjelt K, Krasilnikoff PA, Grauballe PC. Incidence of hospitalization and outpatient clinical visits caused by rotavirus and non-rotavirus acute gastroenteritis. A study of children living in the southern district of Copenhagen County. Dan Med Bull 1984;31:249-51.
6. Matson DO, Estes MK. Impact of rotavirus infection at a large pediatric hospital. J Infect Dis 1990;162:598-604.
7. Visser LE, Cano Portero R, Gay NJ, Martinez Navarro JF. Impact of rotavirus disease in Spain: an estimate of hospital admissions due to rotavirus. Acta Paediatr 1999;88 Suppl 426:S72-6.
8. Rodríguez-Cervilla J, Peñalver MD, Curros MC, Pavón P, Alonso C, Fraga JM. Rotavirus: Estudio clínico y epidemiológico en niños hospitalizados menores de dos años. An Esp Pediatr 1996;45:499-504.
9. Parashar UD, Bresee JS, Gentsch JR, Glass RI. Rotavirus. Emerg Infect Dis 1998;4:561-70.
10. Bernstein DI, Sack DA, Rothstein E, Reisinger K, Smith VE, O'Sullivan D, et al. Efficacy of live, attenuated, human rotavirus vaccine 89-12 in infants: a randomized placebo-controlled trial. Lancet 1999;354:287-90.

### Israeli Spotted Fever Rickettsia (*Rickettsia conorii* Complex) Associated with Human Disease in Portugal

**To the Editor:** Mediterranean spotted fever is endemic in Portugal, where it is a reportable disease with approximately 1,000 new cases per year (1). *Rickettsia conorii* has been thought to be the only pathogenic rickettsia of the spotted fever group in Portugal (2), as well as in the Western Mediterranean area. Another rickettsia in this group, the Israeli spotted fever rickettsia, which belongs to the *R. conorii* complex (3-5), was isolated in 1974 from ticks and humans; however, its distribution appeared to be restricted to Israel (6). We report three cases of rickettsiosis in Portugal caused by Israeli spotted fever rickettsia.

Case 1. A 71-year-old woman was hospitalized with a history of fever (39ºC) for 6 days, headache, and icterus. The influenzalike syndrome was treated with an antipyretic. In the next 4 days, the patient had myalgias, malaise, and mental confusion. Ten hours after being transferred to an intensive care unit, she died with septic shock and multiorgan failure, despite intravenous administration of doxycycline and other antibiotics.

Case 2. A 79-year-old woman, who was previously healthy except for high blood pressure, was hospitalized with a 4-day history of gastrointestinal disorders, nausea, and vomiting, which were attributed to food poisoning; high fever (40ºC) developed, and 3 days later a cutaneous rash, which spread to the palms and soles. The diagnosis of Mediterranean spotted fever was made by indirect immunofluorescent assay against *R. conorii* (immunoglobulin [Ig] M 1:40; IgG 1:512). The patient was treated with doxycycline and was discharged from the hospital 20 days after admission.

Case 3. A 65-year-old woman was hospitalized with a 6-day history of fever (39ºC), headache, vomiting, and epigastric pain, which had been treated with penicillin. Rash and icterus developed, and the patient died of shock and multiorgan failure 9 hours after hospitalization, despite treatment with a mixture of antibiotics, which contained doxycycline.

Rickettsiae of the spotted fever group were isolated by the shell vial technique from the blood of the three patients. Sequences of polymerase chain reaction-amplified fragments of 16SrRNA (1440 bp), citrate synthase (382 bp), and rompA (590 bp) genes of the isolates show 100% similarity with the homologous sequence of Israeli spotted fever rickettsia (4,7,8).

All three patients lived in semirural areas, along the River Tejo (Setubal District). None had left Portugal during the previous year. Although none had a tache noire, contact with ticks cannot be excluded. The absence of tache noire is typical in Israeli spotted fever (6). These findings indicate that the geographic distribution of Israeli spotted fever is wider than had been thought and includes the Iberian Peninsula. Because initial signs and symptoms of the disease are particularly uncharacteristic and appropriate treatment may be delayed, this rickettsia can cause life-threatening disease.

**Fatima Bacellar,* Lorenza Beati,† Ana França,‡ José Poças,§ Russell Regnery,† and Armindo Filipe***
*Centro de Estudos de Vectores e Doenças Infecciosas, Instituto Nacional de Saúde Dr. Ricardo Jorge, Aguas de Moura, Portugal; †Centers for Disease Control and Prevention, Atlanta, Georgia, USA; ‡Hospital Garcia da Orta, Almada, Portugal; and §Hospital São Bernardo, Setubal, Portugal

### References

1. Tavares L, Botas J, Antunes F, Araújo FC. A febre escaro-nodular em Portugal. O Médico 1985;113:838-46.
2. Bacellar F, Regnery RL, Nuncio S, Filipe AR. Genotypic evaluation of Rickettsial isolates recovered from various species of ticks in Portugal. Epidemiol Infect 1995;114:169-78.
3. Regnery RL, Spruill CL, Plikaytis BD. Genotypic identification of *Rickettsiae* and estimation of interspecies sequence divergence for portions of two rickettsial genes. J Bacteriol 1991;173:1576-89.
4. Fournier PE, Roux V, Raoult D. Phylogenetic analysis of spotted fever group *Rickettsiae* by study of the outer surface protein rOmpA. Int J Sys Bacteriol 1998;48:839-49.
5. Roux V, Raoult D. Phylogenetic analysis and taxonomic relationships among the genus *Rickettsia*. In: *Rickettsiae* and rickettsial diseases at the turn of the third millennium. Raoult D, Brouqui P, editors. Paris: Elsevier; 1999. p. 52-66.
6. Goldwasser RA, Steiman Y, Klingberg W, Swartz TA. The isolation of strains of *Rickettsiae* of the spotted fever group in Israel and their differentiation from other members of the group by immunofluorescence methods. Scand J Infect Dis 1974;6:53-62.
7. Roux V, Raoult D. Phylogenetic analysis of the genus *Rickettsia* by 16S rDNA sequencing. Res Microbiol 1995;146:385-96.
8. Roux V, Rydkina E, Eremeeva M, Raoult D. Citrate syntase gene comparison, a new tool for phylogenetic analysis and its application for *Rickettsiae*. Int J Sys Bacteriol 1997;47:252-61.

### Avoiding Misdiagnosis of Malaria: A Novel Automated Method Allows Specific Diagnosis, even in the Absence of Clinical Suspicion

**To the Editor:** We report three cases of malaria to illustrate a novel method that allows diagnosing the disease, even if clinicians do not suspect it or request malaria smears. Lack of clinical suspicion is a well-known factor for malaria misdiagnosis and may be responsible for almost 40% of deaths from *Plasmodium falciparum* infections in industrialized countries (1-3). A recent study from Canada showed that in 59% of cases malaria was initially misdiagnosed, and in 16% three or more physician contacts occurred before malaria smears were ordered (4).

Early diagnosis of malaria relies crucially on clinical suspicion. A clinician suspecting the disease has to explicitly request malaria smears. This problem has not been solved with the advent of several methods alternative to microscopy, including recently introduced rapid dipstick tests (5). Performing any of these tests blindly without a specific request is impractical. On the other hand, routinely performed laboratory tests in the work-up of febrile patients, e.g., automated full blood counts, have so far detected only nonspecific changes, such as anemia or thrombocytopenia, which are associated with many other conditions (6). These changes on their own are therefore not specific enough to trigger malaria smears without an explicit request.

New automated full blood counts-analyzers incorporate flow-cytometric principles. The Cell-Dyn 3500 (Abbott, Santa Clara, CA) uses scattered laser-light of leukocytes at four different angles to generate a white-blood-cell differential (7). Monocytes and neutrophils may

